# Use of Sacubitril/valsartan in patients with cardio toxicity and heart failure due to chemotherapy

**DOI:** 10.1186/s40959-020-00078-4

**Published:** 2020-11-05

**Authors:** Vanesa Gregorietti, Teresa Lopez Fernandez, Diego Costa, Elías Ortega Chahla, Andrés J. Daniele

**Affiliations:** 1 Cardio-Oncology Department, Roffo Institute, 5481 Av San Martin. CABA,, Buenos Aires, Argentina; 2 Cardiology Department, La Paz University Hospital, Madrid, Spain

**Keywords:** Heart failure, Cancer, Cardiotoxicity, Sacubitril/valsartan

## Abstract

**Background:**

Cancer therapy-related cardiac dysfunction (CTRCD) is a critical problem with an impact on both oncological and cardiovascular prognosis, especially when it prevents patients from receiving cancer treatment. Standard therapy for heart failure (HF) is recommended for CTRCD, but there is no well-established evidence on how sacubitril/valsartan may help cancer patients with cardiotoxicity.

**Objectives:**

The aim of this trial was to study the effectiveness of sacubitril-valsartan in patients with CTRCD treated in cardio-oncology units.

**Methods:**

We enrolled 635 patients with breast cancer and followed them with echocardiography and NT- proBNP. Patients who developed left ventricular dysfunction and heart failure were treated with angiotensin-converting enzyme inhibitors (ACEI) (enalapril) or angiotensin receptor blockers (ARB) (valsartan), aldosterone antagonists (eplerenone), digitalis and diuretics (furosemide), as needed. When patients remained symptomatic and met the PARADIGM-HF inclusion criteria, sacubitril/valsartan was started instead of enalapril or valsartan.

We analyzed clinical, laboratory and echocardiographic variables to determine the beneficial effects of sacubitril/valsartan on left ventricular remodeling (improvement of left ventricular ejection fraction (LVEF), left ventricle internal diameter in diastole), diastolic dysfunction (E/e’ ratio), reduction in NT-proBNP levels, New York Heart Association (NHYA) class and improvement in the 6-min walk test.

Also, we analyzed serum creatinine and potassium levels to determine treatmentsafety in this population. Median follow-up was 20 months.

**Results:**

Twenty-eight patients developed cardiotoxicity and were treated with sacubitril/valsartan. The sacubitril/valsartan dose was 100 mg (sacubitril 49 mg/valsartan 51 mg) in 12 patients (42.85%) and 200 mg (sacubitril 97 mg/valsartan 103 mg) in 16 patients (57.15%). No deaths were reported, and one patient underwent heart transplantation.

Baseline median NT-proBNP was 997.5 pg/ml (IQR 663.8 — 2380.8), which decreased to a median of 416.5 pg/ml (IQR 192.0–798.2) on follow-up with *p* < 0.001.

Baseline NYHA functional class was III (78.6%) or IV (21.4%), and it improved to I (57.1%) or II (42.9%) on follow-up. LVEF increased with treatment from 26.7 ± 5.4% to 32.3 ± 5.5% (*p* < 0.001). There were also significant improvements in left ventricle internal diameter in diastole (LVIDD), diastolic function, 6-min walk test, and mitral valve regurgitation. There were no differences between basal and follow-up levels of serum creatinine or potassium.

**Conclusion:**

Sacubitril/valsartan might be a promising treatment option in patients with refractory CTRCD.

## Background

Heart failure is defined as a constellation of symptoms and signs that are attributed to the inability of the heart to produce a cardiac output meeting body demands. Despite advances in treatment, it is still a life-threatening disease, affecting between 1 and 2% of the population, and is more commonly seen in the elderly, with a prevalence of 6–10% of patients over 65 years [1]. There are different causes of heart failure, and one of them is cancer therapy-related cardiac dysfunction (CTRCD).

CTRCD poses an obstacle in the treatment of cancer patients and is a serious problem for the healthcare system. Its incidence varies according to the cancer therapy analyzed. Classically CTRCD was described as secondary to anthracycline therapy but currently, various oncology therapies (anti HER2, tyrosine kinase inhibitors, proteasome inhibitors) are potential causes [2].

Standard pharmacological therapies indicated for the treatment of HF have been shown to be also useful in CTRCD.

Sacubitril/valsartan (LCZ-696) is a combined neprilysin inhibitor and angiotensin AT1 receptor blocker approved in recent years for the treatment of chronic heart failure with reduced ejection fraction. The PARADIGM-HF study was the trial that led to the approval of the use of sacubitril/valsartan for the treatment of HF [3]. In the PARADIGM-HF study, the subgroup of patients with CTRCD was not evaluated. In an area where there have been limited pharmacological advances in the last 10 years, this drug was a game-changer.

The optimal use of sacubitril/valsartan in clinical practice needs further investigation, in particular for patients with CTRCD as they are usually poorly represented in clinical trials. This trial is the first prospective study that attempts to demonstrate the usefulness of sacubitril/valsartan in the treatment of CTRCD**.**

## Methods

635 consecutive patients with a history of breast cancer who received treatment with chemotherapy and were referred from the Cardio-oncology Department were enrolled prospectively from June 2016 to January 2018.

Cardiovascular evaluation was clinically performed using echocardiography and complete biochemical work-up (including NT-proBNP) at baseline and during treatment for up to 24 months. Patients receiving anthracyclines were followed using the Mayo Clinic protocol [4] and in those treated with anti-HER2 therapies, an echocardiography was performed every 3 months.

We define CTRCD as a reduction in left ventricular ejection fraction (LVEF) of more than 10% from baseline to a LVEF< 53% [5]. We did not have access to strain imaging echocardiography.

The patients who developed cardiotoxicity were treated with beta-blockers (carvedilol), angiotensin-converting enzyme inhibitors (enalapril) or angiotensin receptor blockers (valsartan), aldosterone antagonist (eplerenone) digitalis and diuretics (furosemide) as needed. When patients remained symptomatic and met the PARADIGM-HF inclusion criteria, sacubitril/valsartan was started instead of enalapril or valsartan.

All patients started with a dose of 100 mg (sacubitril 49 mg/valsartan 51 mg) per day and adjustments were performed gradually according to tolerance.

We analyzed clinical, laboratory and echocardiographic variables to determine the beneficial effects of sacubitril/valsartan on left ventricular remodeling (improvement of LVEF, left ventricle internal diameter in diastole), diastolic dysfunction (E/e’ ratio), reduction in NT-proBNP levels, New York Heart Association (NYHA) class and improvement in the 6-min walk test.

Also, we analyzed serum creatinine and potassium levels to determine the treatment’s safety in this population.

### Statistical analysis

Continuous variables were summarized as means ± standard deviations (SD) or medians with interquartile ranges (IQR) and compared with Student’s t-test or Wilcoxon’s test for paired samples, according to their distribution. Categorical values were expressed as percentages and compared with the chi-squared test. Finally, correlations were assessed with Pearson’s correlation coefficient (PCC). Statistical significance was considered with a *p*-value of less than 0.05. All analyses were performed with R version 3.6.1.

## Results

Fifty-one patients developed CTRCD; 28 patients were available for analysis because they met the inclusion criteria. Most of them were women treated for breast cancer with doxorubicin/cyclophosphamide or trastuzumab/pertuzumab.

There was no difference in the cumulative dose of doxorubicin among patients receiving sacubitril/valsartan or not.

The baseline characteristics are outlined in Table 1. The median time from the start of anti-cancer therapy to the occurrence of heart failure with reduced ejection fraction (HFrEF) was 183.6 ± 95.4 days and the median time from HFrEF to sacubitril/valsartan initiation was 48.4 ± 12.3 days.

**Table 1:** Population baseline characteristics

Characteristic (***n*** = 28)	Mean ± SD or n (%)
Age (years)	56.2 ± 13.4
Females	25 (89.3%)
BP	14 (50%)
Smoker	12 (42.86%)
Hypercholesterolemia	10 (35.71%)
Diabetes Mellitus	8 (28.57%)
History of ischemic cardiac disease	2 (7.14%)
Left Breast Cancer	18 (64.3%)
Radiotherapy	9 (32.1%)
Ejection fraction (%)	26.68 (5.35)
Creatinine (mg/dl)	1.31 ± 0.16
*NYHA functional classification*	
I	0 (0%)
II	0 (0%)
III	22 (78.6%)
IV	6 (21.4%)
*Chemotherapy*	
Doxorubicin	23 (82.1%)
Cyclophosphamide	23 (82.1%)
Docetaxel	3 (10.7%)
Trastuzumab	8 (28.6%)
Pertuzumab	4 (14.3%)
Days since chemotherapy started	183.6 ± 95.4
Heart Failure treatment at enrollment	
Beta Blockers	26 (92,85%)
ACEI	12 (42.85%)
ARB	13 (46.43%)
Digitalis	9 (32.14%)
Mineralocorticoids antagonist	18 (64.28%)
Diuretics	20 (71.43%)
ICD	3 (10.71%)
CRT-D	2 (7.14%)
Heart transplant	1 (3.6%)

The dose of sacubitril/valsartan was 100 mg (sacubitril 49 mg/valsartan 51 mg) in 12 patients (42.85%) and 200 mg (sacubitril 97 mg/valsartan 103 mg) in 16 patients (57.15%). One patient received a heart transplant; three, an implantable cardioverter defibrillator, two underwent cardiac resynchronization therapy, and no deaths were reported.

**Fig. 1:**
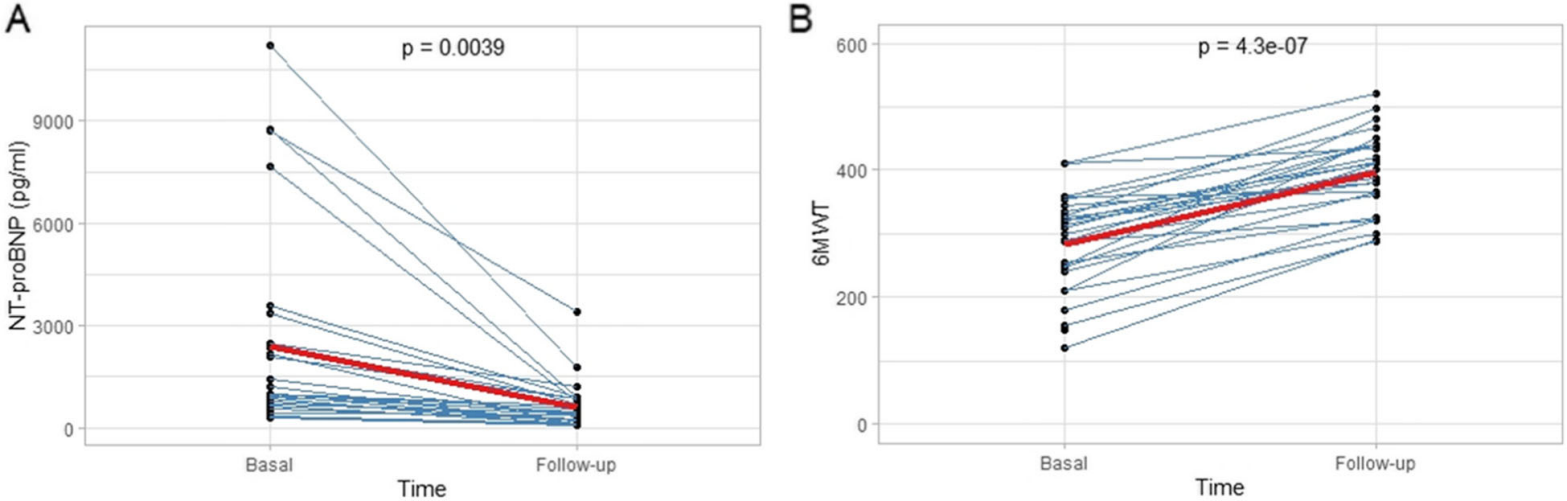
NT-proBNP levels and 6-min walk test change over time. **a***: NT-proBNP individual values and mean change over time.* **b***: 6-min walk test (6MWT) individual values and mean change over time*

Baseline median NT-proBNP was 997.5 pg/ml (IQR 663.8–2380.8). On follow-up, it decreased to a median of 416.5 pg/ml (IQR 192.0–798.2), with *p* = 0.0039. Baseline median 6-min walk test was 300 m (IQR 244.5–332.5), and it increased on follow-up to 410 m (IQR 362.5–440.5), with *p* < 0.001 (Fig. 1).

**Fig. 2:**
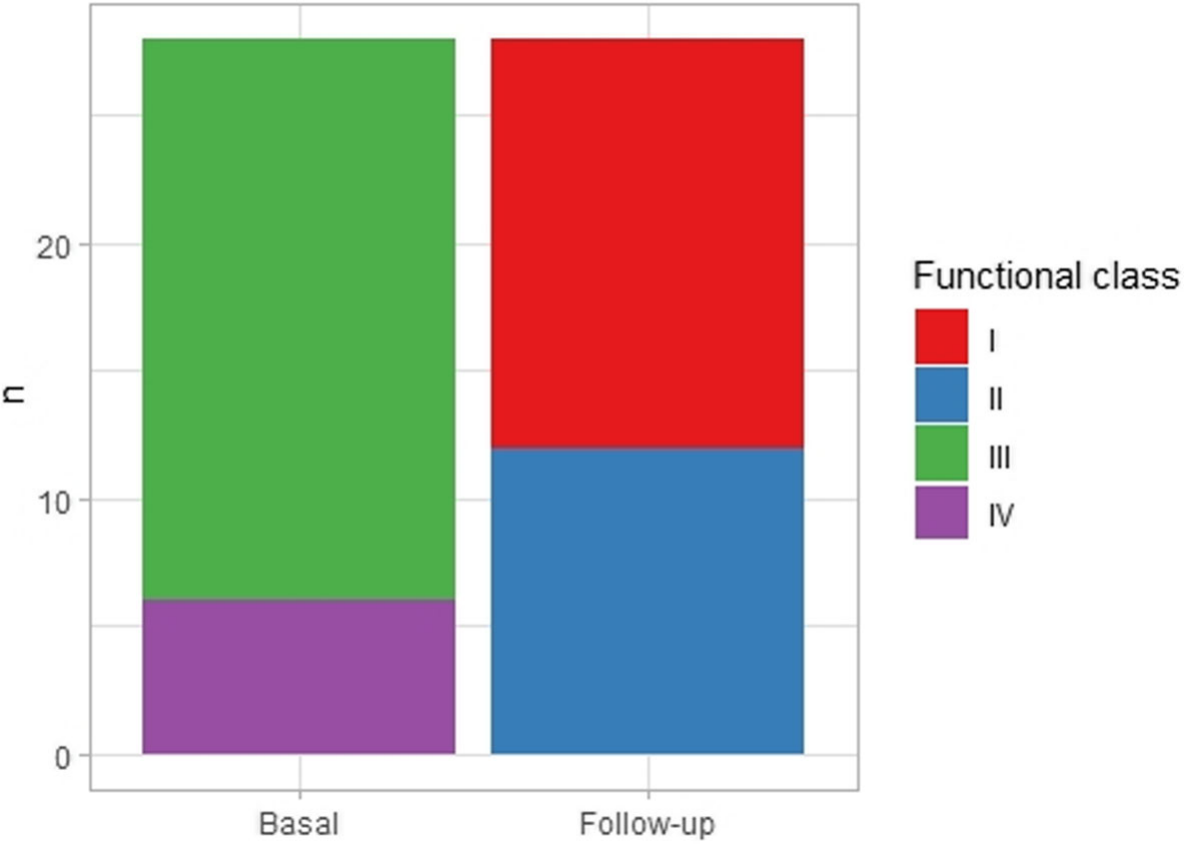
NYHA functional class change over time

When comparing NYHA functional class, the patients were all in class III (78.6%) or IV (21.4%) on baseline, and I (57.1%) or II (42.9%) on follow-up, as seen in Fig. 2. Similar results were obtained when comparing chemotherapy subgroups and considering the time since chemotherapy was started.

When assessing echocardiographic parameters, we found that ejection fraction improved with treatment from 26.7 ± 5.4% to 32.3 ± 5.5% (*p* < 0.001). Additionally, baseline left ventricle internal diameter in diastole (LVIDD) was 67.5 mm (65.00–69.25) and went down to 60 mm (59.00–61.25) after treatment (*p* < 0.001). Diastolic function, as assessed with the ratio between early mitral inflow velocity and mitral annular early diastolic velocity (E/e’) with a cut-off of 15, was reduced from 75% before treatment to 42.9% after treatment (*p* = 0.027). Mitral regurgitation also improved, from a baseline of 21.4% mild, 60.7% moderate, and 17.9% severe, to values after treatment of 67.9, 32.1, and 0.0%, respectively.

**Fig. 3:**
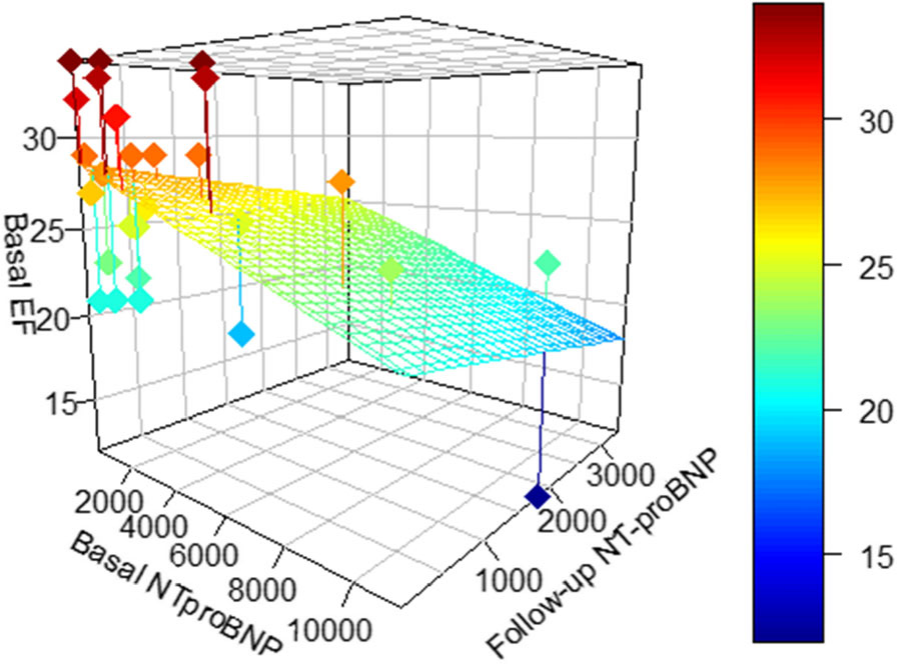
Correlation between NT-proBNP decrease in pg/ml and baseline ejection fraction (EF) in %

There was a negative and significant negative correlation between the decrease in NT-proBNP levels over time and basal ejection fraction (PCC = − 0.44, p 0.019). As evidenced in Fig. 3, the impact on NT-proBNP levels seems to be higher in patients with lower baseline ejection fraction.

There was no difference between basal and follow up levels of creatinine (1.31 ± 0.16 mg/dl vs 1.34 ± 0.17 mg/dl, *p =* ns) or potassium serum (4.1 ± 0.3 mEq/l vs 4 ± 0.2 mEq/l, *p =* ns).

## Discussion

Cardiovascular disease accounts for between 30 and 40% of global mortality in the general population. Heart failure, the final stage of many cardiovascular conditions, is one of the most common causes of morbidity and mortality, representing about 62% of the global cardiovascular deaths, and is growing daily [6].

In the oncological population, ventricular dysfunction and heart failure cause significant limitations in treatment strategies and therefore have a considerable impact on prognosis.

Since the publication of the PARADIGM-HF study, sacubitril/valsartan has been a useful tool for the treatment of patients with ventricular dysfunction and heart failure [3].

Some studies showed improvement of the NYHA class and 6-min walk test with sacubitril/valsartan in patients with HF of etiologies other than CTRCD [7]. Improvement in functional mitral insufficiency after initiation of treatment with sacubitril/valsartan has also been reported [8, 9].

None of these studies included patients with cancer and CTRCD, so its usefulness in this group has now become a question to be answered. Several reports on the use of sacubitril/valsartan in CTRCD have been published in recent years. Many of them constitute case reports [10–12]. In recent years some abstracts have been presented at international congresses evaluating the benefit of sacubitril/valsartan in cancer patients.

The importance of oncological patients completing their specific therapy creates the need to improve the treatment of heart failure in this subgroup of patients in order to meet this goal [13, 14].

Based on these data we conducted our study, the first prospective trial to determine the usefulness of the sacubitril/valsartan in the treatment of CTRCD.

Our study showed that sacubitril/valsartan therapy in patients with CTRCD produces an improvement in ventricular remodeling (recovery of LVEF and ventricular diameters), diastolic dysfunction (E/e’ ratio), and of the symptoms reflected in the NYHA class and the 6-min walk test.

Although we identified a difference between patients who received CRT-D and those who did not, due to the small size of the sample, it was not statistically significant.

On the other hand, the analysis of plasma creatinine levels, glomerular filtration rate, and plasma potassium levels showed that in this type of patients, with their co-existing comorbidities, the use of sacubitril/valsartan is safe.

The evidence suggests that sacubitril/valsartan is a valid option in the treatment of CTRCD patients, but a multicenter study with a larger number of patients would be needed to reach more definitive conclusions.

## Conclusion

Sacubitril/valsartan has shown a good safety profile with excellent follow-up results in patients with refractory left ventricular dysfunction related to chemotherapy, which is a promising option in this patient population.

### Limitations of investigation

The use of sacubitril/valsartan seems promising according to the evidence in this limited group of patients, but a large-scale study should be carried out with a larger number of patients to prove the real usefulness of this drug in cardiac failure with ventricular dysfunction due to cardiotoxicity secondary to chemotherapy.

### Competencies in medical knowledge

In breast cancer patients with ventricular dysfunction associated with cardiotoxicity and refractory heart failure, the use of sacubitril-valsartan has proven to be an excellent treatment option in this specific population.

### Translational outlook

More research is needed with a larger number of patients with ventricular dysfunction and heart failure due to cardiotoxicity to evaluate the usefulness of sacubitril-valsartan in this particular group of patients.

## Data Availability

Not applicable.

## Abbreviations

CTRCD: Cancer Treatment Caiac Dysfunction
HF: Heart Failure
HFrEF: Heart Failure reduced Ejection Fraction
ACEI: Angiotensin-converting enzyme inhibitors
ARB: Angiotensin receptor blockers
LVEF: Left Ventricular Ejection Fraction
NYHA: New York Heart Association
LVIDD: Left Ventricular Internal Diameter in Diastole
